# Vaccination or mass drug administration against schistosomiasis: a hypothetical cost-effectiveness modelling comparison

**DOI:** 10.1186/s13071-019-3749-4

**Published:** 2019-10-23

**Authors:** Benjamin S. Collyer, Hugo C. Turner, T. Déirdre Hollingsworth, Matt J. Keeling

**Affiliations:** 1 Zeeman Institute (SBIDER), Mathematics Institute, University of Warwick, Coventry, UK; 2 Oxford University Clinical Research Unit, Wellcome Trust Overseas Programme, Ho Chi Minh City, Vietnam; 3 Centre for Medicine and Global Health, Nuffield Department of Medicine, University of Oxford, Oxford, UK; 4 Big Data Institute, Li Ka Shing Centre for Health Information and Discovery, University of Oxford, Oxford, UK; 5 School of Life Sciences, University of Warwick, Coventry, UK

**Keywords:** Schistosomiasis, Vaccine, Cost-effectiveness, Modelling

## Abstract

**Background:**

Schistosomiasis is a neglected tropical disease, targeted by the World Health Organization for reduction in morbidity by 2020. It is caused by parasitic flukes that spread through contamination of local water sources. Traditional control focuses on mass drug administration, which kills the majority of adult worms, targeted at school-aged children. However, these drugs do not confer long-term protection and there are concerns over the emergence of drug resistance. The development of a vaccine against schistosomiasis opens the potential for control methods that could generate long-lasting population-level immunity if they are cost-effective.

**Methods:**

Using an individual-based transmission model, matched to epidemiological data, we compared the cost-effectiveness of a range of vaccination programmes against mass drug administration, across three transmission settings. Health benefit was measured by calculating the heavy-intensity infection years averted by each intervention, while vaccine costs were assessed against robust estimates for the costs of mass drug administration obtained from data. We also calculated a critical vaccination cost, a cost beyond which vaccination might not be economically favorable, by benchmarking the cost-effectiveness of potential vaccines against the cost-effectiveness of mass drug administration, and examined the effect of different vaccine protection durations.

**Results:**

We found that sufficiently low-priced vaccines can be more cost-effective than traditional drugs in high prevalence settings, and can lead to a greater reduction in morbidity over shorter time-scales. MDA or vaccination programmes that target the whole community generate the most health benefits, but are generally less cost-effective than those targeting children, due to lower prevalence of schistosomiasis in adults.

**Conclusions:**

The ultimate cost-effectiveness of vaccination will be highly dependent on multiple vaccine characteristics, such as the efficacy, cost, safety and duration of protection, as well as the subset of population targeted for vaccination. However, our results indicate that if a vaccine could be developed with reasonable characteristics and for a sufficiently low cost, then vaccination programmes can be a highly cost-effective method of controlling schistosomiasis in high-transmission areas. The population-level immunity generated by vaccination will also inevitably improve the chances of interrupting transmission of the disease, which is the long-term epidemiological goal.

## Background

Schistosomiasis is estimated to affect over 250 million people, primarily in sub-Saharan Africa and South America. It is caused by water-borne parasitic flukes from the genus *Schistosoma* (predominantly *Schistosoma mansoni*, *Schistosoma haematobium* and *Schistosoma japonicum)*, which enter the body through the skin and colonize the host’s bloodstream. Mated *Schistosoma* produce fertilized eggs which stimulate an immune response, and this can lead to multiple pathologies including stunted growth, anemia, and in cases of severe burden, fibrosis of internal organs [1]. Eggs are passed into environmental water systems through excreta, where they hatch and asexually multiply through intermediary snail hosts, completing their life-cycle.

Control in endemic regions is through mass drug administration (MDA), using the drug praziquantel [2]. Currently, MDA is implemented predominantly through school-based initiatives targeting school-aged children (SAC), although in some areas community-wide programmes that also target adults are employed [3]. Substantial progress has been made recently in widening coverage, and schistosomiasis is on course to reach its WHO 2020 control target of treating 75% of SAC in endemic regions. Despite these advances, schistosomiasis is failing to meet the 2020 WHO control target of reducing heavy-intensity infections to below 5% prevalence in endemic regions [4]. Also, evidence demonstrating the ability of MDA to control the transmission of schistosomiasis in high prevalence areas is mixed, in part because the impact of MDA will vary across different epidemiological settings. There are many regions, such as the Mekong River in Cambodia, where excellent progress has been made, with heavy-intensity infections reduced to below 1% [5, 6]. However, several recent studies in Africa have demonstrated limited progress in reducing prevalence in localized high-transmission areas, despite high MDA coverage [7–9]. Mathematical modelling suggests that high coverage of both children and adults over sustained periods of time is required for MDA to control schistosomiasis in high-transmission areas, which may prove to be beyond practical limits [10, 11].

Vaccines against schistosomiasis are under development, the most promising of which utilize the antigen protein Sm-p80. These vaccines are highly effective in baboon models: reducing the rate of establishment, fecundity and life-span of colonizing *S. mansoni*, particularly female worms [12–18]. The potential benefits for patients of an efficacious vaccine with lasting protection are clear; however, before investing in human trials, it is important to consider value-for-money in comparison to existing control methods. Studies conducted 20 years previously, when an earlier generation of vaccine candidates were being investigated, used deterministic models to compare the effect of vaccination to MDA, and estimated the cost-effectiveness of vaccination but without modelling the transmission [19, 20]. In this paper, we developed an individual-based model of schistosomiasis infection and control which accounts for population level heterogeneity and dynamical complexity. The model is used to investigate the cost-effectiveness of a potential vaccine across a range of plausible scenarios, varying the transmission setting, vaccine characteristics and delivery programmes. Also, by benchmarking the cost effectiveness of vaccination programmes against the cost effectiveness of current MDA programmes, we calculate a critical vaccination cost beyond which vaccination may not be economically favorable.

## Methods

### Transmission model

Our analysis uses a stochastic individual-based transmission model, where *S. mansoni* are explicitly transmitted between a population of human hosts and an external reservoir. This type of modelling for human helminth infections, first utilized by Anderson & Medley, means that considerable biological heterogeneity can be incorporated and allows for detailed examination of potential control strategies [21]. Since their inception, substantial improvements in computational power have allowed the use of individual-based models to efficiently sample their resulting distributions over time and overcome the inherent noise in the dynamics. An alternative approach using deterministic compartment-based models, has been used to investigate the effects of vaccination, but this approach allows for less scope for including biological complexity [22, 23]. The transmission cycle, which is related to previously published models [11, 21, 24], is as follows:

- Mature adult schistosomes reproduce within human hosts monogamously. To account for within-host competition, fecundity (egg production per female schistosome) decays exponentially with the total number of mature schistosomes currently contained in the human host.
- Eggs are passed into an external reservoir, after which they hatch into larval stages. In the reservoir, the larval stages have a fixed probability of dying each day. We do not model the snail component of the life-cycle explicitly, because the dynamics of processes within the snails are fast in comparison to those within human hosts, so we may consider the density of larvae in the environment to a local equilibrium determined by the within human-population burden. Alternative models that include the snail component have been used to investigate the effect of molluscicides on the transmission [25, 26].
- Each day, human hosts come into contact with the reservoir and are infected. The average number of infecting larvae is proportional to the density of larvae in the reservoir, the individual’s risk-factor (assigned at birth from a gamma distribution) and a function of individual’s age. We assume that the population has generally poor access to sanitation, and hence the risk-factor and age only influence the uptake of larvae, not the deposition of eggs into the environment [27]. When a larval stage has infected a human host, it matures into an adult and is randomly assigned a sex.

Our model was parameterized with previously published values and to match published epidemiological studies (see Table 1 and Additional file 1: Figure S1).

**Table 1:** Parameter values used in our individual-based transition model, and their sources

Parameter	Value	Reference
No. of human hosts in population, *N*	1000	
Baseline prevalence in SAC	High-transmission setting, 65%; Moderate-transmission setting, 45% (Obtained by scaling the contact rate between human hosts and the reservoir)	
Age-dependent contact with reservoir	Ages 0–4: 0.032; 5–9: 0.162; 10–14: 1.0; 15+: 0.05	[43]
Shape parameter α of Gamma distributed risk factor	0.24	[43]
Kato–Katz test aggregation parameter, *k*	0.87	[44, 45]
Exponential fecundity decay, *z*	0.0007/worm	[23, 46]
Maximum fecundity, λ	0.14 egg counts/female worm	[45, 47]
Average worm life-span	5.7 years	[48]
Human demography	Based on Kenya’s demographic profile	[49]

### Prevalence measurement

For the implementation of control programmes, WHO guidelines require prevalence levels in the community to be monitored [3]. The Kato–Katz [28, 29] fecal smear test is the most widely used diagnostic test for *S. mansoni*. Eggs are counted by eye from two samples to give an average number eggs per gram. Egg counts between 0 and 4 indicate a low intensity infection, between 4 and 16 indicate a moderate intensity infection, and greater than 16 indicate a heavy intensity infection [30]. We accounted for the over-dispersed nature of the recorded egg counts [31, 32] using a negative-binomial distribution. We stress that all results presented are in terms of this realized egg count, and not in terms of true worm-burden; this is important for vaccination which suppresses egg output, and because morbidity is most closely correlated with egg output.

### Interventions: vaccination

The final characteristics of a human vaccine against schistosomiasis are as yet unknown, hence we used our mathematical model to consider the implications of different vaccine attributes (focusing on duration of protection) and different patterns of deployment. Immunization of an individual is likely to be achieved with multiple vaccine doses spread across a number of weeks [14], dependent on vaccine characteristics and logistical factors. Given this uncertainty, we made the simplifying assumption that immunization occurs instantaneously at a specified time point. This assumption should have limited effect on results, because the period over which the vaccine is delivered is expected to be short compared to the duration of immunity.

On successful immunization, the vaccine is assumed to have two effects on the epidemiology of schistosomes: (i) a reduction of establishment of schistosomes in human hosts; and (ii) a reduction in fecundity of mature female schistosomes.

In keeping with recent trials of Sm-p80 vaccines in baboons, we made three key assumptions about the vaccine [14]. We assumed the vaccine does not have a therapeutic effect on already established schistosomes, i.e. there is no increase in the death rate. We modelled a partially efficacious vaccine that reduces establishment of new worms by 90% and reduces fecundity by 90%, which is comparable to the efficacy of a Sm-p80 vaccine in baboon model trials, although the duration of protection remains uncertain and is a key sensitivity in all our predictions. To cover a range of different potential vaccine protection durations, we generated results for vaccines with protection that lasts 2.5, 5, 10 and 20 years.

We modelled two types of vaccination programme: cohort delivery and delivery to larger groups. Cohort delivery is implemented by vaccinating children in particular age-groups annually. The age-groups chosen are based on the duration of vaccine protection, so that immunity is maintained up to age 15 (Table 2). Further to this, we modelled the option of implementing a catch-up campaign in the first year of the programme to ensure than the whole target age-groups has protection from the first year of intervention. When modelling synchronized delivery to larger groups, such as school-aged children (SAC) (with coverage the same as MDA), the vaccine is administered either once every two years or once every five years depending on the duration of vaccine protection.

**Table 2:** Schedule for cohort vaccination. Coverage for each age is 70%

Vaccine protection duration	Cohort ages
2.5 years	1, 3, 5, 7, 9, 11, 13
5 years	1, 6, 11
10 years	1, 11
20 years	1

### Interventions: mass drug administration

The only drug widely available for preventative chemotherapy for schistosomiasis is praziquantel (PZQ), which is effective against all *Schistosoma* species [1]. WHO guidelines recommend a minimum coverage of at least 75% of at-risk school-aged children [3]. However, in practice this level of coverage is not often attained [33], so our results are produced with three different coverage levels: (i) 40%; (ii) 60%; and (iii) 75%. Further to this, we included a scenario where community-based delivery is modelled, with a SAC coverage of 75% and adult coverage of 40%, which previous modelling suggests is a coverage level that is able to break transmission in a high-prevalence setting [34]. PZQ is assumed to kill 86.3% of adult schistosomes within human hosts, but has no long-lasting impact on either establishment or fecundity [35].

Systematic non-compliance, whereby individuals have a propensity to partake in MDA or not, can create a reservoir of untreated hosts and will reduce the efficacy of MDA strategies. We modelled this using the methodology of Dyson et al. [36], where the correlation of attendance between rounds is controlled independently to the coverage. We used a correlation parameter $\rho = 0.4$ which is consistent with the range of correlations found for MDA within multiple studies (see references in [36]).

### Cost and cost-effectiveness analysis

Cost-effectiveness requires both a quantification of the health benefits of a given treatment programme as well as the associated economic costs. To measure the benefit of applying an intervention, we counted the total number of days that each individual has a heavy-intensity infection (HII), as measured by the Kato–Katz test [30] and recorded the total heavy-intensity infection years averted relative to the baseline of no intervention over a 30 year time horizon. Heavy-intensity infections have been chosen as this accounts for the majority of negative health and societal effects (although we acknowledge significant schistosomiasis-related conditions such as anemia occur even for light-infections, and present alternative results where health benefits are measured in infection years averted in Additional file 1: Figures S2, S3) [37].

The cost efficacy of vaccination relative to MDA strategies is critically dependent on the relative prices of vaccination and administering PZQ. One of the great benefits of MDA is that the drugs and delivery are relatively cheap; the assumed costs of MDA are outlined in Table 3, using data from Additional file 1: Tables S1, S2. The costs for MDA delivery were assumed proportional to the number targeted for treatment (and not the number subsequently treated); while the cost for PZQ tablets was directly dependent on how many were treated.

**Table 3:** Economic and financial costs for MDA delivery, obtained from online WHO regression tool [51] using median input values (see Table 4), and mean GDP per capita weighted by population needed to treat across endemic countries (see Additional file 1: Table S2)

Cost	Economic costs		Financial costs		Reference
	School-based delivery	Community-wide delivery	School-based delivery	Community-wide delivery	
Delivery cost (per person targeted)	US$0.75	US$0.50^a^	US$0.31	US$0.34	[52]
PZQ tablets	2.5 × US$0.08 per child	2.5 × US$0.08 per child; 3.5 × US$0.08 per adult	Donated	Donated	[53, 54]

*Notes*: Economic costs include costs associated with Ministry of Health buildings and time, as well as the value associated with volunteers’ time

^a^Includes US$0.08 per person for the opportunity costs related to the community volunteer who distribute the drugs which is not included with the WHO regression tool [52]

The costs of the vaccination programme are unknown, so we performed two pieces of analysis. First, we considered three different immunization costs: US$3, US$6 and US$12 per full course of vaccine (not per dose and including delivery); this provides a more natural way of comparing vaccines that may require different number of doses. Secondly, we considered the critical vaccination cost, which is the cost of a course of vaccine that leads to the same cost-effectiveness as MDA (targeted at the appropriate equivalent section of the population). This can be considered to be a maximum price one would be willing to pay for vaccination, if cost is the only deciding factor.

Both analyses were performed under the health care providers perspective and quantify the cost-effectiveness of an intervention by the HII years averted per US$ spent over the course of the intervention. As recommended by the WHO, a discount rate of 3% per annum was applied to both the costs and effects [38].

## Results

### Comparison of interventions

**Fig. 1:**
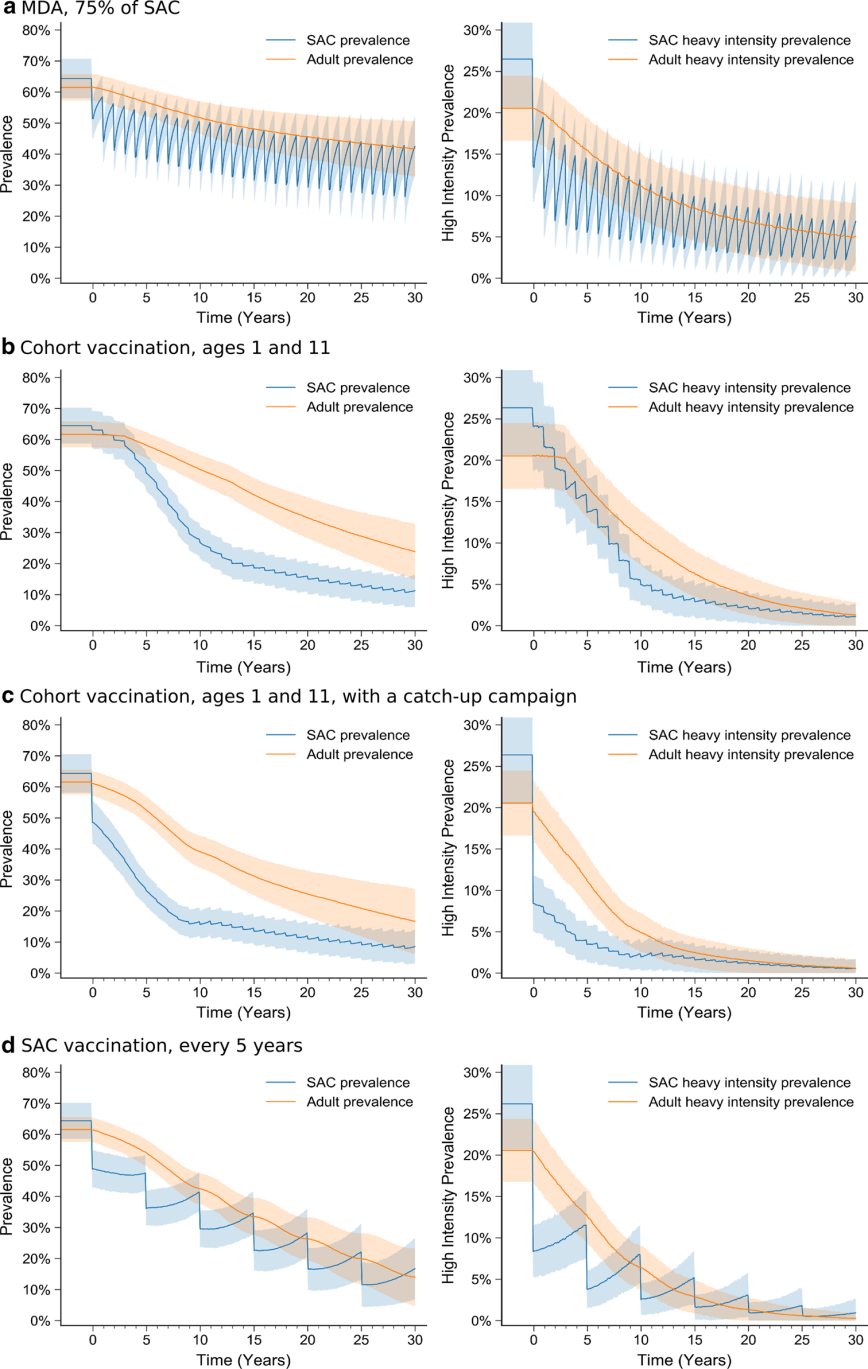
High-transmission setting: prevalence (left) and heavy intensity infection prevalence (right) in SAC and adults during 30 years of control. Shaded regions represent the 95% prediction interval (i.e. 95% of all stochastic simulations lie within this region). **a** MDA targeted at SAC with 75% coverage. **b** Cohort vaccination (at 1- and 10 years-old). **c** Cohort vaccination (at 1- and 10 years-old) and a catch-up campaign in the first year. **d** Mass SAC vaccination, every 5 years. In all cases the vaccine is assumed to offer 10 years protection

Figure 1a shows the time-series of prevalence and heavy intensity infection prevalence in SAC (blue) and adults (orange) during 30-years of MDA targeted at SAC, starting from a high-transmission baseline. The ‘bounce-back’ effect, where recently treated hosts are rapidly re-infected, prevents large reductions in prevalence, despite years of repeated treatment.

We contrast this with a vaccine that provides protection for ten years, delivered to children in cohorts (at age 1 and 10 years); this generates a greater reduction in prevalence and heavy intensity prevalence in both SAC and adults than MDA (Fig. 1c). The reduction happens in two phases: (i) over the first ten year there is a reduction in SAC prevalence, due to increasing proportions of immunized children; and (ii) after ten years the whole SAC class is protected, and subsequent reductions in prevalence due to a declining environmental reservoir are slower. The ultimate goal of interrupted transmission is not achieved within 30 years.

When a catch-up campaign is added in the first year of intervention (targeting individuals aged 1–15 years), prevalence and heavy-intensity infection prevalence are rapidly reduced (Fig. 1c), although there is a more limited impact on the longer-term prevalence (compare Fig. 1b and 1c). When SAC are vaccinated once every 5-years (Fig. 1d) there is a notable bounce-back after intervention, as unvaccinated pre-SAC children mature into the SAC group, but this is much slower than the bounce-back seen after a round of MDA. The long-term prevalence is comparable to that seen in the cohort strategies.

**Fig. 2:**
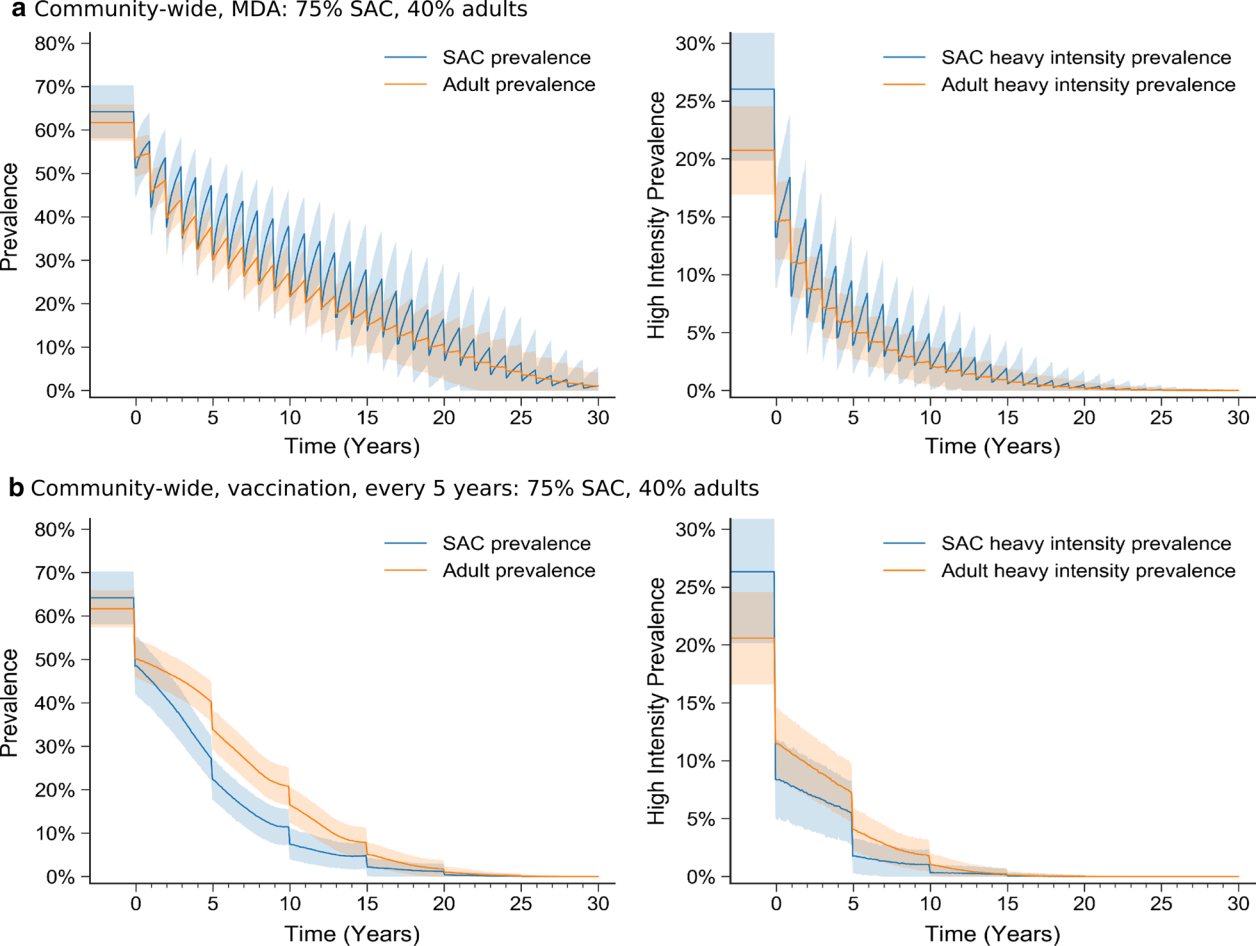
High-transmission setting: prevalence (left) and heavy intensity infection prevalence (right) in SAC and adults during 30 years of control. **a** MDA targeted at the whole community (75% SAC coverage, 40% adult coverage). **b** Vaccination every 5 years, with a vaccine that offers 10 years protection, targeted at the community (75% SAC coverage, 40% adult coverage)

Broader interventions that target the community are able to give faster and greater reductions in prevalence (Fig. 2). After 20-years of community-wide MDA treatment (75% SAC, 40% adult coverage), heavy-intensity infections are effectively eliminated, and within 30 years interruption of transmission is also possible (Fig. 2a). When vaccination is delivered at the whole-community level (again vaccinating 75% SAC and 40% adults), the speed of eradication is faster still, i.e. eradicating heavy intensity infection after 15 years.

### Cost-effectiveness: high-transmission setting

**Fig. 3:**
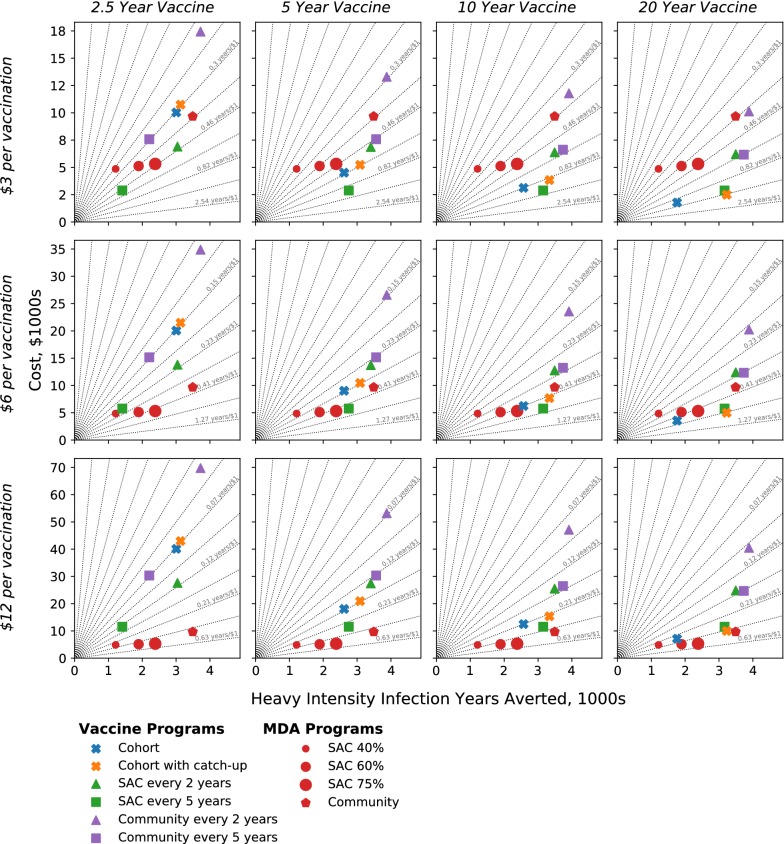
High-transmission setting: incremental cost-effectiveness diagrams across differing vaccine protection lengths (columns) and relative vaccination costs (rows), for MDA and vaccination-based strategies (points). Radial gridlines (grey) indicate equal cost-efficacy (i.e. the same number of heavy-intensity infection years averted per dollar). The cost per vaccination represents the full course of vaccine (not per dose and including delivery)

Figure 3 shows the cost-efficacy of each intervention; columns correspond to differing vaccine durations, rows to differing assumed vaccination costs (US$3, US$6 and US$12 per full course of vaccine including delivery). Each point corresponds to a different strategy and reflects the benefits of the programme (x-axis) against the economic costs (y-axis). Strategies to the right (more effective) and below (cheaper) are more cost-effective than those to the left and above. Grey lines link points with equal cost-effectiveness.

Due to the economies of scale, MDA becomes more cost-effective as coverage increases (red circles for coverages of 40%, 60% and 75% of school-aged children (SAC)). Extending MDA to the whole community (red pentagon) provides much more benefit than SAC MDA, but is less cost-effective; thus while community MDA offers a greater reduction in heavy-infections it comes at a relatively higher cost; a greater reduction would be to distribute the same amount of PZQ to school-aged children in more communities.

Somewhat counter intuitively, the simple cohort-vaccination programme (blue cross) has the greatest health benefit when the vaccine only offers a short duration of protection; this is because, more age-groups have to be vaccinated annually hence protection of all SAC is reached earlier; however, this leads to a much higher cost associated with the programme. For a similar reason including a catch-up campaign is of greater benefit for vaccines with longer protection, although catch-up campaigns are always less cost-effective than the underlying cohort-vaccination (comparing blue and orange crosses). Community vaccination (purple symbols) is associated with high costs, but has the potential to achieve elimination if the vaccine confers sufficiently long immunity. The high costs are partly offset by interruption of transmission (meaning that no further vaccinations are administered from that point onwards), but such gains are limited due to discounting in predicted costs and benefits over long time periods. For vaccines that confer less than 20-years protection, immunizing SAC every 5 years (green square) is the most cost-effective vaccine-based strategy, while immunizing children in cohorts together with a catch-up campaign (orange cross) is the most cost-effective vaccine strategy if protection lasts for 20 years. When comparing the cost effectiveness of vaccination against MDA, the cost of delivering a full course of the vaccine is obviously a key consideration. When the costs are just US$3.0 for immunization (top row of Fig. 3), vaccination can be more cost-effective than MDA, depending on choosing the best deployment strategy. As vaccination costs increase, the most cost-effective method becomes dependent on the assumed duration of protection. However, when the costs are US$12.0 per course of vaccine, MDA targeted at SAC (with at least 75% coverage) is always the most cost-effective intervention regardless of vaccine protection duration.

**Fig. 4:**
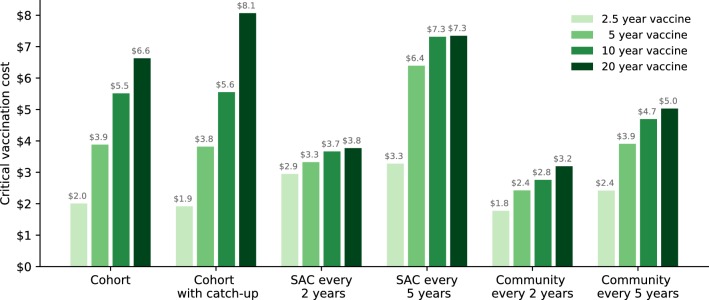
High-transmission setting: critical vaccination costs (comprising delivery and vaccine costs), relative to MDA, for school and community-wide strategies. Critical vaccination cost is defined by the cost, per course of vaccine, that achieves the same cost-effectiveness as MDA targeted at the relevant section of the community

Rather than considering a limited number of vaccination costs, we now calculate a critical vaccination cost (defined as the cost, per course of vaccine, that achieves the same cost-effectiveness as MDA targeted at the relevant section of the community) for each vaccination strategy, and for each vaccine duration of protection (Fig. 4). These critical vaccination costs provide a rapid assessment of the maximal price for vaccination to be preferable to MDA, all other aspects being equal.

For a 2.5-year-duration vaccine, our predictions suggest that costs for vaccination greater than US$3.0 per vaccination are not cost-effective, regardless of strategy. However, as the duration of protection increases, so does the critical vaccination cost. For 5- and 10-year vaccines, vaccinating SAC every 5 years provides the most cost-efficacy with a critical vaccination cost of US$6.5 and US$7.4 per vaccination course, while for vaccines that give 20 years of protection, the cohort based strategies provide the greatest cost-efficacy, leading to a critical vaccination cost of US$8 per vaccination course. Hence, the vaccine must be able to provide long-duration protection and be less than $8 per course for it to be more cost-effective than current MDA control measures. We note that while community-wide vaccination provides the most health benefits, the vaccination of adults provides less efficacy as adults generally have less contact with the infectious reservoir.

### Cost-effectiveness: moderate-transmission setting

**Fig. 5:**
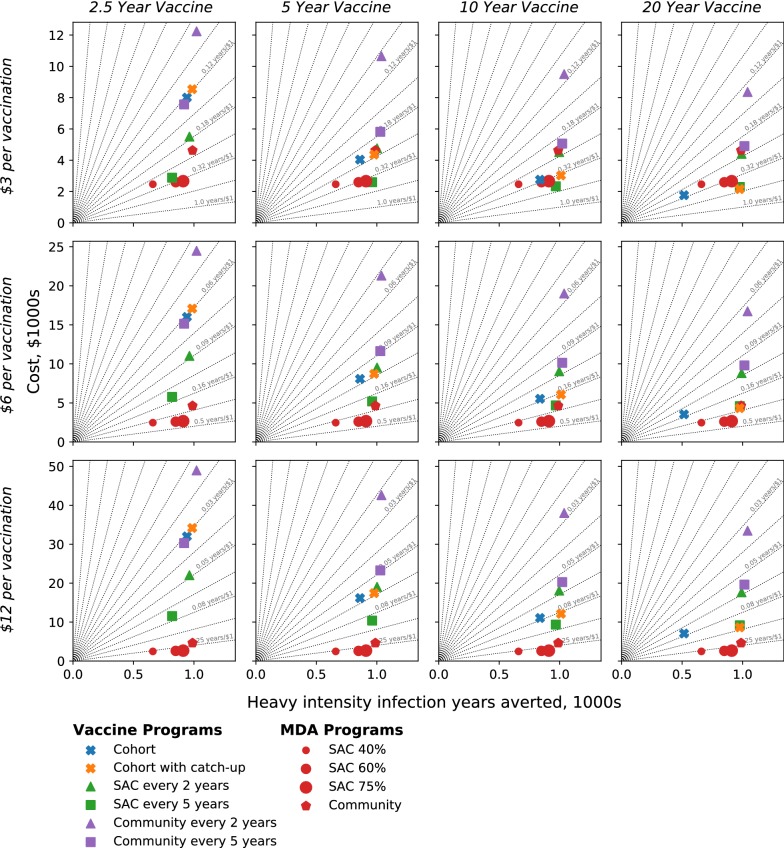
Moderate-transmission setting incremental cost-effectiveness diagrams across differing vaccine protection lengths (columns) and relative vaccination costs (rows), for MDA and vaccination based strategies. Radial gridlines indicate locations on the plane of equal cost-efficacy. The cost per vaccination represents the full course of vaccine (not per dose and including delivery)

**Fig. 6:**
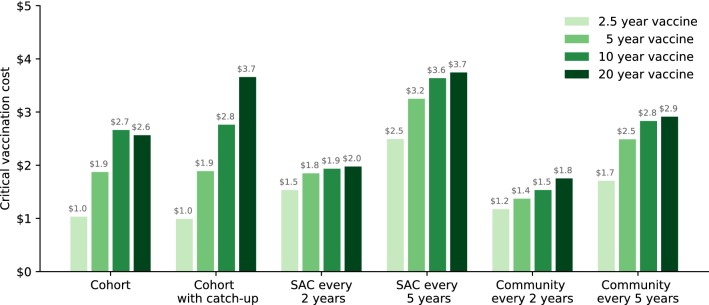
Moderate-transmission setting: critical vaccination costs (comprising delivery and vaccine costs), relative to MDA, for school and community-wide strategies. Critical vaccination cost is defined by the cost, per course of vaccine, that achieves the same cost-effectiveness as MDA targeted at the relevant section of the community

In Figs. 5 and 6 we repeated our analysis in a moderate-transmission setting (with a baseline SAC prevalence of 45%). In general, lower baseline prevalence leads to MDA being relatively more cost-effective compared to vaccination, as the reinfection after chemotherapy is slower (Fig. 5). Many strategies now provide similar levels of reduction of heavy-intensity infections, and so cost-effectiveness is determined purely by the cost of the strategy. Only when the vaccination is extremely cheap (US$3 per course) and protection is long-lasting can it be more cost-effective than MDA. As expected, the critical vaccination cost in the moderate-transmission setting are consistently lower than in the high-transmission setting (Fig. 6). Our modelling indicates that one should only be willing to pay up-to US$3.7 per vaccination course, should cost be the only deciding factor in choosing between MDA and vaccination.

## Discussion

We have developed a predictive individual-based model of schistosomiasis dynamics that can account for the action of both traditional drug treatment and control by vaccines currently under development; this allowed us to assess both the epidemiological impact and associated cost of different control strategies. The model has been matched to data from a variety of epidemiological studies and mechanistically captures the individual-levels dynamics of humans and schistosomes; however, there are a number of facets where additional data could help to refine the model assumptions, as discussed below.

Our model neglects the possible role of acquired immunity that may be caused by prolonged exposure to schistosomes [39]. This could potentially reduce the benefits of a vaccine, as older individuals would already be experiencing some level of immunity. Acquired immunity for schistosomiasis is not well understood, partly due to the difficulty in disentangling its effect from age-related exposure, and further research in this area is needed.

Due to the limited understanding of the causal link between schistosomiasis morbidity and burden, caution should be employed when interpreting modelling results regarding the amount of schistosomiasis morbidity averted by different interventions [37]. In principle, the disability-adjusted life year (DALY) averted would be an ideal metric to use, but further research is needed in this area to make this possible [37].

There are also limited cost data on MDA particularly regarding the relative cost of school *vs* community-based treatment (and therefore the results regarding the relative cost-effectiveness of these interventions should be treated with caution). There is an important research need for improving the economic evaluations of different schistosomiasis interventions.

Finally, our modelling does not include the effect of improvements to water, sanitation and hygiene (often referred to as WASH) that may be expected over the course of a control programme. Including these effects into the modelling relies on the availability of quantitative data that measure both exposure and contribution to the infective reservoir, and at present time this longitudinal data have proved challenging to collect.

The development of a vaccine through the stages of clinical trials will require substantial resources, and the challenge of developing a successful vaccine against a macroparasite should not be underestimated; efforts to find a vaccine for schistosomiasis have been ongoing since the 1990s [40]. The production of a licensed vaccine is at a minimum ten years away, so the need for a vaccine is contingent on the amount of progress being made during that time period. The global trend of increased coverage for preventative chemotherapy is encouraging. As progress continues, the analysis presented in this study should be repeated with models fit to longitudinal data that accurately represent the situations where a vaccine would be used. This may include scenarios where vaccination follows MDA, or MDA and vaccination are used in combination. The impact of such an approach is likely to be sensitive to the initial period of MDA, whether vaccination is used singly or in combination, as well as the unknown characteristics of the vaccine. Such a multi-dimensional exploration of parameter space is beyond the scope of this paper, and would be best to be considered on a case-by-case basis. It should be noted that vaccination in high-transmission areas where prevalence has been suppressed by MDA is not directly comparable to vaccination in un-controlled low and moderate transmission settings, even if the prevalence levels are similar. Current evidence suggests that in high-transmission settings, where the vaccine is most cost-effective, infection may still persist at relatively high levels in ten years’ time even when MDA is applied.

In high transmission settings, we estimated a critical vaccination costs of approximately US$8 per vaccine course if long-duration protection is generated; at this price vaccination and MDA are equally cost-effective. This generates an upper bound on the potential willingness to pay for the vaccine. However, and most importantly, these do not necessarily reflect a realistic or achievable market price for a schistosomiasis vaccine. To put these costs into context, one can take the recent example of vaccines targeted against Human Papillomavirus (HPV), Gardasil and Cervarix, which are produced by Merck and GSK respectively. In 2018 the median price of Gardasil-4 and Cervarix-2 for Gavi-supported countries was US$4.55 per dose. For non-Gavi middle-income countries median prices were approximately US$16 per dose, while in high-income countries the prices for Gardasil-4 and Cervarix-2 were $26 and $38 per dose [41]. Note that Gardasil requires a 2-dose schedule, while Cervarix requires a 3-dose schedule, further increasing the costs, in addition to the costs associated with delivery must also be factored into the calculation. Furthermore, unlike for HPV, there is no market for a schistosomiasis vaccine in high-income countries. The example of HPV would suggest that a significant subsidy is necessary for a vaccine to be produced at a price low enough for the countries which harbour the heaviest schistosomiasis burden to (i) afford to be able to purchase and deliver the quantities required; and (ii) be as cost-effective as MDA campaigns with high coverage.

Importantly, it should be noted that the results generated in this study are for a hypothetical vaccine with a mode of action and efficacy that resembles that of the Sm-p80 vaccine in baboon experiments, and that further analysis should be conducted if a vaccine is developed to the point where efficacy and safety in humans can be measured.

## Conclusions

Given that an estimated 243 million people live in high-risk areas for schistosomiasis, there is a strong need for cheap and effective methods to reduce the burden and associated morbidity [42]. Our cost-effectiveness comparisons of MDA and vaccination are highly dependent on the transmission setting, the duration of vaccine protection and the cost of the vaccine. They are also dependent on the efficacy of the vaccine in humans being similar to the efficacy found in baboon trials. In high-transmission settings, we found that all treatments (both MDA and vaccine) have a substantial impact on schistosomiasis, significantly reducing the prevalence of high worm burdens. In general, well-targeted vaccination campaigns produce a greater reduction in heavy-intensity infections than MDA, although the cost-effectiveness is highly sensitive to the duration of protection generated by the vaccine, the cost of each dose and the transmission setting, i.e. long protection, low cost and high transmission all make vaccines more cost-effective. Our results therefore provide key characteristics for when vaccination is more cost effective than current MDA strategies. Under the most favorable conditions examined (high transmission setting, 20 years of protective immunity and an optimal deployment strategy) vaccination offers substantial health benefits over school-aged MDA but is only more cost-effective than MDA if immunization can be achieved for less than US$8. Vaccines offering shorter immunity are less cost-effective although they can still generate substantial health benefits; while in lower-transmission settings the cost-effectiveness of vaccination relative to MDA is further reduced. Two additional factors, beyond cost effectiveness, may influence the decision to adopt vaccination as a control method. The first is the greater reductions in heavy intensity infections that can be achieved by vaccination. The second is concerns about the potential emergence and spread of drug resistance to praziquantel, in which case vaccination offers an alternative method of control.

## Supplementary information

**Additional file 1: Figure S1.** Egg output data. Normalised mean egg output from our transition model at equilibrium (red, shaded area represents 95% credible interval) and empirical data from Matithini, Kenya [48, 55]. **Table S1.** Worldwide distribution of schistosomiasis; countries requiring preventative chemotherapy (2016) and their GDP per capita for production of weighted mean GDP per capita used to generate costs [56]. **Table S2.** Parameters and values used to estimate MDA programme costs, generated from median values in Fitzpatrick et al. study [50, 52]. GDP per capita value is the mean GDP per capita across countries with endemic schistosomiasis weighted by population (see Table S1). **Figure S2.** High transmission setting: incremental cost-effectiveness diagrams across differing vaccine protection lengths (columns) and relative vaccination costs (rows), for MDA and vaccination-based strategies (points). Radial gridlines (grey) indicate equal cost-efficacy (i.e. the same number of infection years averted per dollar). The cost per vaccination represents the full course of vaccine (not per dose and including delivery). **Figure S3**. High transmission setting: incremental cost-effectiveness diagrams across differing vaccine protection lengths (columns) and relative vaccination costs (rows), for MDA and vaccination-based strategies (points). Radial gridlines (grey) indicate equal cost-efficacy (i.e. the same number of infection years averted per dollar). The cost per vaccination represents the full course of vaccine (not per dose and including delivery).

## Data Availability

Data supporting the conclusions of this article are included within the article. The datasets generated during and/or analysed during the present study are available from the corresponding author upon reasonable request.

## Abbreviations

MDA: mass drug administration
SAC: school-aged children
PZQ: praziquantel
HII: heavy-intensity infection
WHO: World Health Organization

## References

[1] Colley DG, Bustinduy AL, Secor WE, King CH (2014). Human schistosomiasis. Lancet..

[2] Doenhoff MJ, Hagan P, Cioli D, Southgate V, Pica-Mattoccia L, Botros S (2009). Praziquantel: its use in control of schistosomiasis in sub-Saharan Africa and current research needs. Parasitology..

[3] WHO (2011). Helminth control in school-age children: a guide for managers of control programmes.

[4] Uniting to Combat NTDs: Ending Neglected Tropical Diseases: A gateway to Universal Health Coverage, Fifth progress report on the London Declaration on NTDs; 2017.

[5] Croce D, Porazzi E, Foglia E, Restelli U, Sinuon M, Socheat D (2010). Cost-effectiveness of a successful schistosomiasis control programme in Cambodia (1995–2006). Acta Trop..

[6] Khieu V, Sayasone S, Muth S, Kirinoki M, Laymanivong S, Ohmae H (2019). Elimination of schistosomiasis mekongi from endemic areas in Cambodia and the Lao Peopleʼs Democratic Republic: current status and plans. Trop Med Infect Dis..

[7] Phillips AE, Gazzinelli-Guimaraes PH, Aurelio HO, Ferro J, Nala R, Clements M (2017). Assessing the benefits of five years of different approaches to treatment of urogenital schistosomiasis: A SCORE project in northern Mozambique. PLoS Negl Trop Dis..

[8] Wiegand RE, Mwinzi PNM, Montgomery SP, Chan YL, Andiego K, Omedo M (2017). A persistent hotspot of *Schistosoma mansoni* infection in a five-year randomized trial of praziquantel preventative chemotherapy strategies. J Infect Dis..

[9] Elmorshedy H, Bergquist R, Emam Abou N, Eassa S, Elsakka E, Barakat R (2015). Can human schistosomiasis mansoni control be sustained in high-risk transmission foci in Egypt?. Parasit Vectors..

[10] Toor J, Turner HC, Truscott JE, Werkman M, Phillips AE, Alsallaq R (2018). The design of schistosomiasis monitoring and evaluation programmes: the importance of collecting adult data to inform treatment strategies for *Schistosoma mansoni*. PLoS Negl Trop Dis..

[11] Toor J, Alsallaq R, Truscott JE, Turner HC, Werkman M, Gurarie D (2018). Are we on our way to achieving the 2020 goals for schistosomiasis morbidity control using current World Health Organization guidelines?. Clin Infect Dis..

[12] Ahmad G, Zhang W, Torben W, Damian RT, Wolf RF, White GL (2009). Protective and antifecundity effects of Sm-p80-based DNA vaccine formulation against *Schistosoma mansoni* in a nonhuman primate model. Vaccine..

[13] Karmakar S, Zhang W, Ahmad G, Torben W, Alam MU, Le L (2014). Use of an Sm-p80-based therapeutic vaccine to kill established adult schistosome parasites in chronically infected baboons. J Infect Dis..

[14] Siddiqui AJ, Molehin AJ, Zhang W, Ganapathy PK, Kim E, Rojo JU (2018). Sm-p80-based vaccine trial in baboons: efficacy when mimicking natural conditions of chronic disease, praziquantel therapy, immunization, and *Schistosoma mansoni* re-encounter. Ann N Y Acad Sci..

[15] Zhang W, Ahmad G, Le L, Rojo JU, Karmakar S, Tillery KA (2014). Longevity of Sm-p80-specific antibody responses following vaccination with Sm-p80 vaccine in mice and baboons and transplacental transfer of Sm-p80-specific antibodies in a baboon. Parasitol Res..

[16] Zhang W, Molehin AJ, Rojo JU, Sudduth J, Ganapathy PK, Kim E (2018). Sm-p80-based schistosomiasis vaccine: double-blind preclinical trial in baboons demonstrates comprehensive prophylactic and parasite transmission-blocking efficacy. Ann N Y Acad Sci..

[17] Ahmad G, Zhang W, Torben W, Ahrorov A, Damian RT, Wolf RF (2011). Preclinical prophylactic efficacy testing of Sm-p80-based vaccine in a nonhuman primate model of *Schistosoma mansoni* infection and immunoglobulin G and E responses to Sm-p80 in human serum samples from an area where schistosomiasis is endemic. J Infect Dis..

[18] Zhang W, Ahmad G, Torben W, Noor Z, Le L, Damian RT (2010). Sm-p80-based DNA vaccine provides baboons with levels of protection against *Schistosoma mansoni* infection comparable to those achieved by the irradiated cercarial vaccine. J Infect Dis..

[19] Chan MS, Woolhouse MEJ, Bundy DAP (1997). Human schistosomiasis: potential long term consequences of vaccination programmes. Vaccine..

[20] Guyatt HL, Evans D (1995). Desirable characteristics of a schistosomiasis vaccine: some implications of a cost-effectiveness analysis. Acta Trop..

[21] Anderson RM, Medley GF (1985). Community control of helminth infections of man by mass and selective chemotherapy. Parasitology..

[22] Alsallaq RA, Gurarie D, Ndeffo Mbah M, Galvani A, King C (2017). Quantitative assessment of the impact of partially protective anti-schistosomiasis vaccines. PLoS Negl Trop Dis..

[23] Stylianou A, Hadjichrysanthou C, Truscott JE, Anderson RM (2017). Developing a mathematical model for the evaluation of the potential impact of a partially efficacious vaccine on the transmission dynamics of *Schistosoma mansoni* in human communities. Parasit Vectors..

[24] Anderson RM, May RM (1991). Infectious diseases of humans: dynamics and control.

[25] Gurarie D, King CH, Yoon N, Li E (2016). Refined stratified-worm-burden models that incorporate specific biological features of human and snail hosts provide better estimates of *Schistosoma* diagnosis, transmission, and control. Parasit Vectors..

[26] Lo NC, Gurarie D, Yoon N, Coulibaly JT, Bendavid E, Andrews JR (2018). Impact and cost-effectiveness of snail control to achieve disease control targets for schistosomiasis. Proc Natl Acad Sci USA.

[27] Sow S, Polman K, Vereecken K, Vercruysse J, Gryseels B, de Vlas SJ (2008). The role of hygienic bathing after defecation in the transmission of *Schistosoma mansoni*. Trans R Soc Trop Med Hyg..

[28] Katz N, Chaves A, Pellegrino J (1972). A simple device for quantitative stool thick-smear technique in schistosomiasis mansoni. Rev Inst Med Trop Sao Paulo..

[29] WHO (1994). Bench aids for the diagnosis of intestinal parasites.

[30] WHO (2002). Prevention and control of schistosomiasis and soil-transmitted helminthiasis: report of a WHO expert committee.

[31] Booth M, Vounatsou P, Ngoran EK, Tanner M, Utzinger J (2003). The influence of sampling effort and the performance of the Kato–Katz technique in diagnosing *Schistosoma mansoni* and hookworm co-infections in rural Côte dʼIvoire. Parasitology..

[32] Hall A (1981). Quantitative variability of nematode egg counts in faeces: a study among rural Kenyans. Trans R Soc Trop Med Hyg..

[33] Burnim M, Ivy JA, King CH (2017). Systematic review of community-based, school-based, and combined delivery modes for reaching school-aged children in mass drug administration programs for schistosomiasis. PLoS Negl Trop Dis..

[34] Anderson RM, Turner HC, Farrell SH, Yang J, Truscott JE (2015). What is required in terms of mass drug administration to interrupt the transmission of schistosome parasites in regions of endemic infection?. Parasit Vectors..

[35] Zwang J, Olliaro PL (2014). Clinical efficacy and tolerability of praziquantel for intestinal and urinary schistosomiasis-a meta-analysis of comparative and non-comparative clinical trials. PLoS Negl Trop Dis..

[36] Dyson L, Stolk WA, Farrell SH, Hollingsworth TD (2017). Measuring and modelling the effects of systematic non-adherence to mass drug administration. Epidemics..

[37] Turner HC, Truscott JE, Bettis AA, Farrell SH, Deol AK, Whitton JM (2017). Evaluating the variation in the projected benefit of community-wide mass treatment for schistosomiasis: implications for future economic evaluations. Parasit Vectors..

[38] Baltussen RM, Adam T, Tan-Torres Edejer T, Hutubessy RC, Acharya A, Evans DB (2003). Making choices in health: WHO guide to cost-effectiveness analysis.

[39] Woolhouse ME, Taylor P, Matanhire D, Chandiwana SK (1991). Acquired immunity and epidemiology of *Schistosoma haematobium*. Nature..

[40] Bergquist NR, Colley DG (1998). Schistosomiasis vaccine:research to development. Parasitol Today..

[41] WHO (2019). World Health Organization Global market study: HPV vaccines.

[42] Barry MA, Simon GG, Mistry N, Hotez PJ (2013). Global trends in neglected tropical disease control and elimination: impact on child health. Arch Dis Child..

[43] Anderson RM, Turner HC, Farrell SH, Truscott JE (2016). Studies of the transmission dynamics, mathematical model development and the control of schistosome parasites by mass drug administration in human communities. Adv Parasitol..

[44] De Vlas SJ, Gryseels B (1992). Underestimation of *Schistosoma mansoni* prevalences. Parasitol Today..

[45] De Vlas SJ, Gryseels B, Van Oortmarssen GJ, Polderman AM, Habbema JD (1992). A model for variations in single and repeated egg counts in *Schistosoma mansoni* infections. Parasitology..

[46] Chan MS, Guyatt HL, Bundy DA, Booth M, Fulford AJ, Medley GF (1995). The development of an age structured model for schistosomiasis transmission dynamics and control and its validation for *Schistosoma mansoni*. Epidemiol Infect..

[47] Anderson RM, May RM (1982). Population dynamics of human helminth infections: control by chemotherapy. Nature..

[48] Fulford AJ, Butterworth AE, Ouma JH, Sturrock RF (1995). A statistical approach to schistosome population dynamics and estimation of the life-span of *Schistosoma mansoni* in man. Parasitology..

[49] Anderson R, Truscott J, Hollingsworth TD (2014). The coverage and frequency of mass drug administration required to eliminate persistent transmission of soil-transmitted helminths. Philos Trans R Soc Lond B Biol Sci..

[50] Fitzpatrick C, Fleming FM, Madin-Warburton M, Schneider T, Meheus F, Asiedu K, et al. Benchmarks for the cost per person of mass treatment against neglected tropical diseases. https://healthy.shinyapps.io/benchmark/ (2016). Accessed 30 Nov 2018. 10.1371/journal.pntd.0005037 PMC5137870 27918573

[51] Turner HC, Toor J, Bettis AA, Hopkins AD, Kyaw SS, Onwujekwe O (2019). Valuing the unpaid contribution of community health volunteers to mass drug administration programs. Clin Infect Dis..

[52] Fitzpatrick C, Fleming FM, Madin-Warburton M, Schneider T, Meheus F, Asiedu K (2016). Benchmarking the cost per person of mass treatment for selected neglected tropical diseases: an approach based on literature review and meta-regression with web-based software application. PLoS Negl Trop Dis..

[53] Leslie J, Garba A, Boubacar K, Yaye Y, Sebongou H, Barkire A (2013). Neglected tropical diseases: comparison of the costs of integrated and vertical preventive chemotherapy treatment in Niger. Int Health..

[54] Leslie J, Garba A, Oliva EB, Barkire A, Tinni AA, Djibo A (2011). Schistosomiasis and soil-transmitted helminth control in Niger: cost effectiveness of school based and community distributed mass drug administration [corrected]. PLoS Negl Trop Dis..

[55] Fulford AJ, Butterworth AE, Sturrock RF, Ouma JH (1992). On the use of age-intensity data to detect immunity to parasitic infections, with special reference to *Schistosoma-Mansoni* in Kenya. Parasitology..

[56] WHO. WHO PCT Databank. https://www.who.int/neglected_diseases/preventive_chemotherapy/sch/en/ (2018). Accessed Nov 2018.

