# Supplementary material for: Vaccination or mass drug administration against schistosomiasis: a hypothetical cost-effectiveness modelling comparison
Source: Parasit Vectors. 2019 Oct 23;12:499. doi: 10.1186/s13071-019-3749-4 (PMC6813092; doi:10.1186/s13071-019-3749-4)
Supplement: Supplementary file 1 — Additional file 1: Figure S1. Egg output data. Normalised mean egg output from our transition model at equilibrium (red, shaded area represents 95% credible interval) and empirical data from Matithini, Kenya [48, 55]. Table S1. Worldwide distribution of schistosomiasis; countries requiring preventative chemotherapy (2016) and their GDP per capita for production of weighted mean GDP per capita used to generate costs [56]. Table S2. Parameters and values used to estimate MDA programme costs, generated from median values in Fitzpatrick et al. study [50, 52]. GDP per capita value is the mean GDP per capita across countries with endemic schistosomiasis weighted by population (see Table S1). Figure S2. High transmission setting: incremental cost-effectiveness diagrams across differing vaccine protection lengths (columns) and relative vaccination costs (rows), for MDA and vaccination-based strategies (points). Radial gridlines (grey) indicate equal cost-efficacy (i.e. the same number of infection years averted per dollar). The cost per vaccination represents the full course of vaccine (not per dose and including delivery). Figure S3. High transmission setting: incremental cost-effectiveness diagrams across differing vaccine protection lengths (columns) and relative vaccination costs (rows), for MDA and vaccination-based strategies (points). Radial gridlines (grey) indicate equal cost-efficacy (i.e. the same number of infection years averted per dollar). The cost per vaccination represents the full course of vaccine (not per dose and including delivery). [file 13071_2019_3749_MOESM1_ESM.docx]

**Additional file 1**


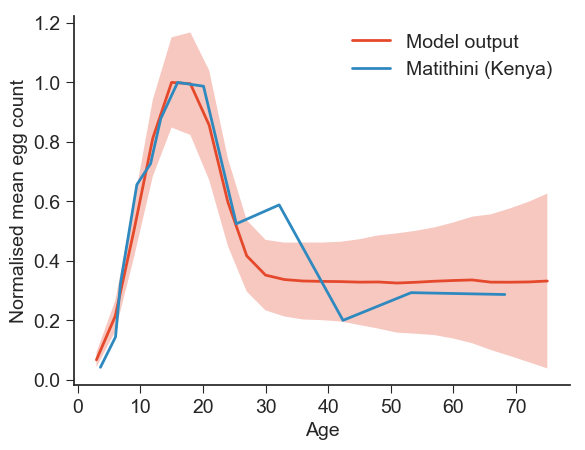


=

**Figure S1 . Egg output data;** normalised mean egg output from our transition model at equilibrium (red, shaded area represents 95% credible interval) and empirical data from Matithini, Kenya (1)

**Table S1. World-wide distribution of schistosomiasis;** countries requiring preventative chemotherapy (2016)(2) and their GDP per capita for production of weighted mean GDP per capita used to generate costs.

| **Country** | **Requiring Preventative Chemotherapy (2016)** | **GDP per capita** | **Country** | **Requiring Preventative Chemotherapy (2016)** | **GDP per capita** |
| --- | --- | --- | --- | --- | --- |
| **Angola** | 5,124,793 | $4,170 | **Liberia** | 1,309,355 | $456 |
| **Benin** | 2,335,801 | $830 | **Madagascar** | 7,803,826 | $450 |
| **Botswana** | 176,687 | $7,596 | **Malawi** | 7,066,970 | $338 |
| **Brazil** | 1,535,838 | $9,821 | **Mali** | 4,692,983 | $824 |
| **Burkina Faso** | 4,130,347 | $671 | **Mauritania** | 589,697 | $1,137 |
| **Burundi** | 2,983,194 | $320 | **Mozambique** | 14,257,757 | $416 |
| **Cambodia** | 76,241 | $1,384 | **Namibia** | 492,527 | $5,227 |
| **Cameroon** | 3,694,802 | $1,447 | **Niger** | 3,517,926 | $378 |
| **Central African Rep.** | 938,761 | $418 | **Nigeria** | 24,246,551 | $1,968 |
| **Chad** | 3,239,149 | $670 | **Philippines** | 2,939,693 | $2,988 |
| **China** | 153,436 | $8,827 | **Rwanda** | 1,779,414 | $748 |
| **Congo** | 164,125 | $1,658 | **Sao Tome and Principe** | 35,678 | $1,913 |
| **Côte d'Ivoire** | 4,235,789 | $1,662 | **Senegal** | 3,278,785 | $1,033 |
| **DR Congo** | 13,309,917 | $458 | **Sierra Leone** | 946,625 | $499 |
| **Egypt** | 173,463 | $2,412 | **Somalia** | 578,279 | $500 |
| **Equatorial Guinea** | 59,143 | $9,850 | **South Africa** | 5,446,352 | $6,161 |
| **Eritrea** | n/a | n/a | **Sudan** | 8,948,450 | $2,899 |
| **Ethiopia** | 15,941,459 | $768 | **Swaziland** | 190,594 | $3,224 |
| **Gabon** | 183,767 | $7,221 | **Togo** | 4,391,622 | $617 |
| **Gambia** | 138,688 | $483 | **Uganda** | 12,352,473 | $604 |
| **Ghana** | 9,604,514 | $1,641 | **Tanzania** | 14,990,824 | $936 |
| **Guinea** | 3,678,503 | $825 | **Venezuela** | n/a | n/a |
| **Guinea-Bissau** | 183,821 | $724 | **Yemen** | n/a | n/a |
| **Indonesia** | 22,675 | $3,847 | **Zambia** | 4,415,815 | $1,510 |
| **Kenya** | 2,484,518 | $1,507 | **Zimbabwe** | 4,044,652 | $1,080 |
| **Laos** | 89,042 | $2,457 | **South Sudan** | n/a | n/a |

**Table S2: Parameters and values used to estimate MDA programme costs**, generated from median values in Fitpatrick et al study (3). GDP per capita value is the mean GDP per capita across countries with endemic schistosomiasis weighted by population (see Supplementary Table S1).

| **Parameter** | **Value** |
| --- | --- |
| National or subnational | Subnational |
| Number of diseases integrated | 1 |
| Number of rounds per year | 1 |
| Year of implementation | 3rd |
| GDP per capita | US$ 1389.93 |
| Population density | 134 per sq. km |
| Coverage rate | 85% |

**Cost-Effectiveness: benefit measured in infection years averted**

We have additionally performed the analysis where the benefit of an intervention is instead measured in infection years averted, as opposed to heavy-intensity infection years averted.


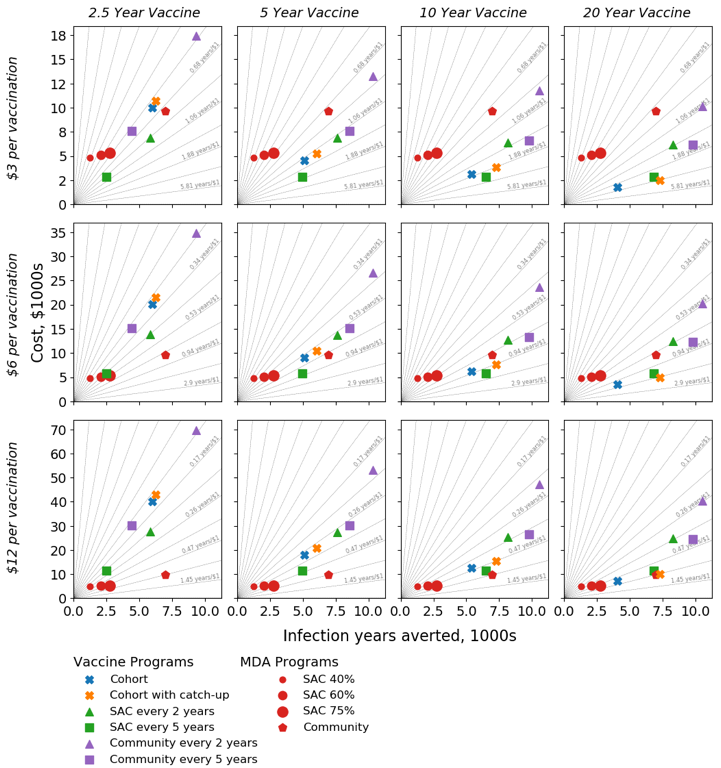


**Figure S2. High transmission setting: incremental cost-effectiveness diagrams** across differing vaccine protection lengths (columns) and relative vaccination costs (rows), for MDA and vaccination based strategies (points). Radial gridlines (grey) indicate equal cost-efficacy (i.e. the same number of infection years averted per dollar). The cost per vaccination represents the full course of vaccine (not per dose and including delivery).

The observed patterns in Figure S2 are very similar to those when the analysis is performed with heavy intensity infection years averted, with the difference that vaccination performs slightly better relative to MDA. Critical vaccination costs (Figure S3) are higher than those found using heavy intensity infections as the measured benefit, because vaccination is directly able to prevent infection whereas MDA is not. When we use infection years averted as our measure of health benefit, a vaccine that costs up to USD$15.7 per vaccination can be as cost-effective as MDA.


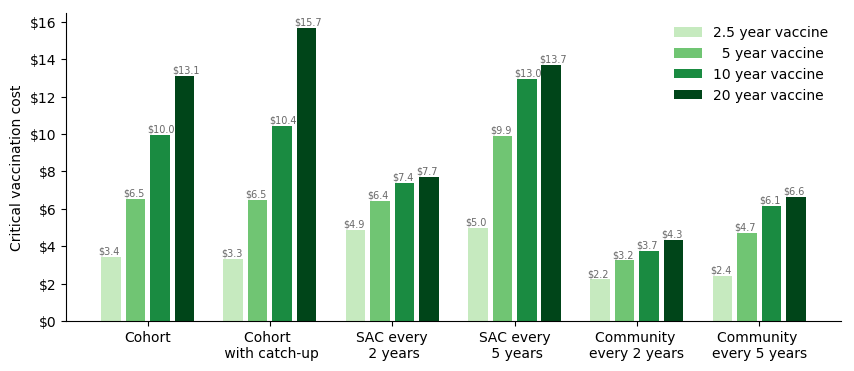


**Figure S3 High transmission setting: incremental cost-effectiveness diagrams** across differing vaccine protection lengths (columns) and relative vaccination costs (rows), for MDA and vaccination based strategies (points). Radial gridlines (grey) indicate equal cost-efficacy (i.e. the same number of infection years averted per dollar). The cost per vaccination represents the full course of vaccine (not per dose and including delivery).

References

1. Fulford AJ, Butterworth AE, Ouma JH, Sturrock RF. A statistical approach to schistosome population dynamics and estimation of the life-span of Schistosoma mansoni in man. Parasitology. 1995;110.

2. WHO PCT Databank 2018 [Available from: <https://www.who.int/neglected_diseases/preventive_chemotherapy/sch/en/>.

3. Fitzpatrick C, Fleming FM, Madin-Warburton M, Schneider T, Meheus F, Asiedu K, et al. Benchmarking the Cost per Person of Mass Treatment for Selected Neglected Tropical Diseases: An Approach Based on Literature Review and Meta-regression with Web-Based Software Application. PLOS Neglected Tropical Diseases. 2016;10(12):e0005037.
